# A refined method for multivariate meta-analysis and meta-regression

**DOI:** 10.1002/sim.5957

**Published:** 2013-08-29

**Authors:** Daniel Jackson, Richard D Riley

**Affiliations:** a MRC Biostatistics Unit, Institute of Public HealthCambridge, U.K.; b Department of Public Health, University of BirminghamBirmingham, U.K.

**Keywords:** multivariate meta-analysis, multivariate *t* distribution, random effects models, small sample inference

## Abstract

Making inferences about the average treatment effect using the random effects model for meta-analysis is problematic in the common situation where there is a small number of studies. This is because estimates of the between-study variance are not precise enough to accurately apply the conventional methods for testing and deriving a confidence interval for the average effect. We have found that a refined method for univariate meta-analysis, which applies a scaling factor to the estimated effects’ standard error, provides more accurate inference. We explain how to extend this method to the multivariate scenario and show that our proposal for refined multivariate meta-analysis and meta-regression can provide more accurate inferences than the more conventional approach. We explain how our proposed approach can be implemented using standard output from multivariate meta-analysis software packages and apply our methodology to two real examples. © 2013 The Authors. Statistics in Medicine published by John Wiley & Sons, Ltd.

## 1. Introduction

Meta-analysis is the pooling of the results from separate studies concerned with the same treatment or issue, and multivariate meta-analysis 1,2 is becoming more commonly used. Multivariate meta-analysis is used to synthesise multiple outcome effects whilst allowing for their correlation. By making use of multiple outcomes in the same analysis, multivariate meta-analysis can provide better inference, but this comes at the price of making more assumptions 3.

The standard approach 3 for making inferences using the random effects model for multivariate meta-analysis, like its univariate counterpart, treats all variance components as fixed and known when pooling the study results. In practice, however, both the within and between-study covariance matrices must be estimated. If the studies are sufficiently large, then taking the within-study covariance matrices as known is an acceptable approximation. Taking the between-study covariance matrix as known however requires a reasonably large number of studies to provide precise estimates of the between-study variance components. Typically meta-analyses involve quite small numbers of studies, and often the most pressing concern when applying the standard methods is that there are too few studies to justify the usual approach.

Hartung and Knapp 4,5 and Sidik and Jonkman 6 proposed a refined method for univariate random effects meta-analysis. As in the more conventional method, this involves estimating the between-study variance and then taking this as known. However, the refined method made further calculations, where a scaling factor is applied to the variance of the pooled estimate, which provides a justification for using a *t*, rather than a standard normal, distribution for making inferences. The refined method provides exact inference for the special case where all studies are the same ‘size’ (same within-study variances) 7, so it is not surprising that the refined method results in better inference than the standard method 8, which does not 9. Simulation studies 4–6 show that the refined method also provides more accurate inference in small samples when the variances differ, but more generally, it too is only approximate.

Because the refined method has been found to provide more accurate inference in situations where there are few studies, obvious questions are ‘can the refined method be extended to the multivariate scenario?’ and ‘if so, does the refined multivariate method offer any advantages?’ In this paper, we show that the answer to both these questions is ‘yes’. In Section 2, we briefly describe the refined method for univariate random effects meta-analysis, and in Section 3, we show how to extend this method multivariately. In Section 4, we perform a simulation study that shows that the refined method offers some advantages in situations where there are a small number of studies, and in Section 5 we apply our proposed methodology to two real examples. We conclude with a discussion in Section 6.

## 2. A refined method for univariate meta-analysis

Hartung and Knapp 4,5 and Sidik and Jonkman 6 proposed a refined method for univariate meta-analysis. In this section, we briefly describe this refined method.

The univariate random effects model assumes that the outcome from the *i*th study, *i* = 1,2, … *n*, is distributed as 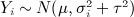, where the within-study variances 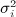 are treated as fixed and known but are estimated in practice. The parameter *μ* is the average outcome or effect and is the parameter of primary interest, and *τ*^2^ is the between-study variance. If *τ*^2^ = 0, then the model is referred to as a fixed effect (common underlying effect across all studies) model, but otherwise between-study heterogeneity is present.

We can obtain the estimate 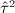 in a variety of ways 8,10,11; the choice of the estimator of *τ*^2^ provides variants of both the conventional and refined methods. In the conventional procedure, the estimator 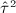 is ‘plugged in’ as the true value when making inferences about the average effect. The estimated average effect is 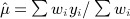, where 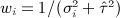 and Var(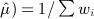. Confidence intervals and hypothesis tests immediately follow using the conventional procedure by assuming that 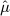 is normally distributed. This now standard procedure does not however take into account the uncertainty in 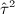, which can be very considerable in small samples. This can have unfortunate implications for the resulting statistical inference, in that actual significance levels and coverage probabilities of confidence intervals may not be very close to their nominal levels.

The refined method 4–6,12 initially follows the conventional procedure 8 by estimating *τ*^2^; some accounts of the univariate refined method 5,6 only mention DerSimonian and Laird′s estimator 8, but others are possible. The refined method involves further calculating what is now referred to as the *H*^2^ heterogeneity statistic 13 where the weights of the study outcomes in this calculation are 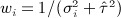 instead of the more usual 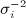. If all variances (both within and between studies) are taken as known then

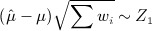
1 where *Z*_1_ denotes a univariate standard normal distribution; (1) is the usual pivotal statistic used for making inference about *μ* in the conventional approach. Continuing to take all variances as known, we also have (but see Copas’ critique 14 of result (2) that follows and the response of Sidik and Jonkman 7)

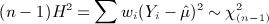
2 where 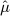 in (1) and *H*^2^ in (2) are independent, so that

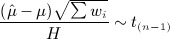
3 Hartung and Knapp 4,5 and Sidik and Jonkman 6 suggested using (3), instead of the more conventional (1), to make inferences about *μ*. They obtained the same point estimate of *μ* as in the conventional approach, but they used an alternative procedure to calculate confidence intervals and perform statistical tests. A comparison of equations (1) and (3) shows that the effect of the refined procedure is to use the conventional approach where Var(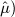 is multiplied by the ‘scaling factor’ *H*^2^ (or equivalently its standard error is multiplied by *H*) and quantiles from the *t* distribution with (*n* − 1) degrees of freedom are used instead of those from a standard normal. The scaling of the variance of the pooled estimate by *H*^2^ is closely related to the method of ‘unconstrained averaging’, as used by Particle Data Group 15.

Knapp and Hartung 12 showed how to extend the refined method to univariate meta-regression and also suggested the possibility of a further *ad hoc* modification where their scaling factor *H*^2^ is constrained to be greater than or equal to one. We develop a refined method for multivariate meta-regression in the next section and so incorporate the refined univariate methods for meta-analysis 4–6 and meta-regression 12 as special cases of our methodology.

## 3. A proposal for a refined method for multivariate meta-analysis and meta-regression

We present the general case for random effects multivariate meta-regression and so include meta-analysis as a special case where there are no study level covariates and intercepts alone are included in the model. We continue to let *n* denote the number of studies, and now let *d* denote the dimension (the number of study outcomes under consideration) of the meta-analysis or meta-regression.

The multivariate random effects meta-regression model 16,17 is

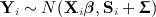
4 for *i* = 1,2, … *n*, where **Y**_*i*_ is the *d* × 1 vector of outcomes (or summary effect measures) associated with study *i*,**S**_*i*_ is the *d* × *d* corresponding within-study covariance matrix, ***Σ*** is the *d* × *d* between-study covariance matrix, **X**_*i*_ is the *d* × *p* design matrix for study *i* and ***β*** is the *p* × 1 vector of true effects. As in the univariate case, we regard the entries of **S**_*i*_ as fixed constants when performing the meta-analysis or regression. If a study does not provide all outcomes, then assuming these are missing at random, the model for the outcomes for study *i* is taken as the marginal model from (4). For a multivariate meta-analysis, for example, where study *i* provides all outcomes, **X**_*i*_ is the *d* × *d* identity matrix and ***β*** is the *d* × 1 average outcome or effects vector. For a multivariate meta-regression, the number of covariate effects that can reasonably be included in the model, and hence the size of *p*, depends on the amount of data available. However, the question of how many covariates may reasonably be included in a multivariate meta-regression model, for a given sample size, is an open question. In the multivariate meta-regression simulation study, in Section 4.4, we consider the bivariate case where *n* = 10, and there is a single covariate effect for both outcomes. This requires estimating two covariate effects on the basis of 20 study outcomes and gives an indication of the minimum amount of information we feel is necessary to reliably fit multivariate meta-regression models in practice.

Just as in the univariate case, a variety of estimates of the between-study covariance matrix are available 3,18–20. We follow the univariate procedures for making inferences about the overall effect as described in the previous section, and the conventional multivariate procedure, in ‘plugging in’ the estimate 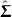. This results in straightforward inference for ***β*** because then all variance components are treated as known.

Let **Y** denote the stacked vector of the observed entries of **Y**_*i*_ and let **X** denote its design matrix. Let Var(**Y**) = ***Δ***^ − 1^, where ***Δ*** incorporates both the within and estimated between-study variance components; ***Δ*** is regarded as fixed and known because all variance components are treated as such. Then

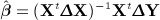


which is approximately normally distributed with covariance matrix

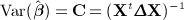


The conventional procedure for making inferences about ***β*** uses the multivariate generalisation of (1),

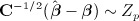
7 where *Z*_*p*_ denotes a standard multivariate normal distribution of dimension *p*. However, as in the univariate case, this does not take into account the uncertainty in the estimate of ***Σ***. A multivariate generalisation of (2), continuing to regard all variance components as known, is

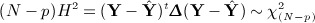
8 where *N* is the total number of estimates (if there are no missing outcome data then *N* = *nd*), 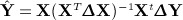 (so that 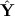 is the ‘fitted vector’ of **Y**), and 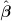 in (7) and *H*^2^ in (8) are independent. Hence, the multivariate generalisation of (3) is

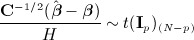
9 where *H*^2^ is now given by (8), *t*(**R**)_(*N* − *p*)_ denotes a central multivariate *t* distribution, with correlation matrix **R** and degrees of freedom (*N* − *p*), and **I**_*p*_ denotes the *p* × *p* identity matrix. Quite a few forms of multivariate *t* distributions exist 21, but this form, which is characterised by the ratio of a multivariate normal distribution and the square root of an independent scalar following a *χ*^2^ distribution, is most common 21; this distribution is described in chapter 1 of Kotz and Nadarajah 22 and, for example, is implemented by the R *mvtnorm* package 23 using the ‘shifted’ option.

Our proposed refined method for multivariate meta-analysis and meta-regression uses (9) for making inferences, whereas the conventional procedure uses (7). As in the univariate case, this means that the same point estimates are obtained as in the conventional method, but an alternative approach is used to calculate confidence regions and perform statistical tests. To use our refined method, we must use the properties for the multivariate *t* rather than the standard normal, distribution. Nadarajah and Kotz 21 provided a good review of the properties of the multivariate *t* distribution, and useful textbooks include Tong 24, ch. 9 (for a good introduction) and Gupta and Nagar 25, ch. 4, where the multivariate *t* distribution is presented as a special case of the matrix variate *t* distribution 25, p. 132.

### 3.1. Making inferences for a single parameter

Standard meta-analytic software 16,26 uses the conventional approach to provide inference for each of the parameters that comprise ***β*** by computing their estimates, standard errors, confidence intervals and giving the results from statistical tests. The proposed method provides the same point estimates as the conventional approach; we can obtain confidence intervals and the results from tests almost as easily using our proposed method and the properties of the multivariate *t* distribution in Sections 10 and 11 of Nadarajah and Kotz 21. A special case of the result in their Section 10 states that if **T** ∼ *t*(**R**)_*v*_ and **B** is a non-singular constant matrix, then

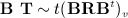
10 The result given in equation (10) is also a consequence of Theorem 4.3.5 of Gupta and Nagar 25.

Furthermore, writing **T** = (** T**_1_,** T**_2_)^*t*^ and

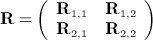


then a special case of the result in Section 11 of Nadarajah and Kotz states that

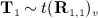
12 Tong 24 gave the marginal distribution in (12) as Proposition 9.1.4.

Results (9), (10) and (12) imply that, denoting the *j*th entries of 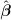 and ***β*** by 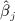 and *β*_*j*_ respectively,

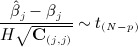
13 where *t*_(*N* − *p*)_ denotes a univariate *t* distribution with (*N* − *p*) degrees of freedom and **C**_(*j*,*j*)_ denotes the entry in the *j*th row and column of **C**. Equation (13) is used for calculating confidence intervals and performing tests concerning a single parameter using our proposed method. The form of (13) shows that the effect of the the refined procedure is to refine the conventional procedure where we multiply Var(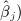 by the scaling factor *H*^2^ (or its standard error by *H*) and use quantiles from the *t* distribution with (*N* − *p*) degrees of freedom when calculating confidence intervals and performing tests, in an analogous way to the univariate case.

More specifically, for example, a 95% confidence interval for *β*_*j*_ is given by

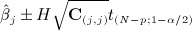
14 where *t*_(*N* − *p*;1 − *α* / 2)_ is the (1 − *α* / 2) quantile of a univariate *t* distribution with (*N* − *p*) degrees of freedom.

### 3.2. Making joint inferences for multiple parameters

Although not provided directly by standard meta-analytic software 16,26, joint inferences for multiple parameters can be obtained using the conventional methodology. For example, a confidence ellipsoid for all parameters can be obtained from

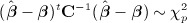
15 so that ***β*** that provides 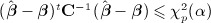 lies within the confidence ellipsoid, where 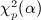 is the *α* quantile of the 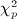 distribution and *α* is the confidence probability. A confidence ellipsoid for a subset of the parameters can be obtained in a similar way to (15) where 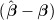 is replaced by a subvector, **C** is the corresponding covariance matrix of reduced dimension and *p* is replaced by the dimension of the subvector of ***β*** that inference is required for.

A very similar approach can be used with the proposed refined method using the result in Section 13 of Nadarajah and Kotz 21 which implies that if **T** ∼ *t*(**I**_*p*_)_(*N* − *p*)_ then **T**^*t*^**T** / *p* ∼ *F*_*p*,(*N* − *p*)_, where *F*_*a*,*b*_ denotes a *F* distribution with *a* and *b* degrees of freedom. Applying this result to (9) gives

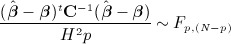
16 so that a confidence ellipsoid using the refined method can be obtained in a similar way. We can obtain a confidence ellipsoid for a subset of the parameters where 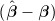 in (16) is replaced by a subvector, **C** is the corresponding covariance matrix of reduced dimension and *p* on the left hand side of (16) is replaced by *p*_1_ where *p*_1_ is the dimension of the subvector of ***β*** that inference is required for. The distribution on the right hand side of (16) then becomes 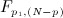.

### 3.3. Making inferences for a linear combination of effects

There may also be interest in making inferences about a linear combination of the estimated effects, indeed the ability to do so is one of the advantages of the multivariate approach 3. This is easily performed using the conventional procedure because multivariate normality is assumed, but this is only very slightly more difficult using our proposed method. We again require the property in Section 10 of Nadarajah and Kotz 21, which says that a linear function of a multivariate *t* distribution is itself multivariate *t* with the same degrees of freedom. A consequence of this result is that when making inferences about a linear combination of effects using the refined procedure, we can again follow the conventional procedure, but as before, we scale the resulting variance of the estimated linear combination by *H*^2^ and use quantiles from a *t* distribution with (*N* − *p*) degrees of freedom rather than a standard normal distribution when making inferences. This procedure includes the method for making inferences for a single parameter in Section 3.1 as a special case.

### 3.4. Inference assuming that all studies have the same within-study covariance matrix, provide all outcomes and there are no covariates

It is important to check that the proposed method performs well for this artificial special case, in part, because of the exchange between Copas 14 and Sidik and Jonkman 7. If all within-study covariance matrices are the same, then **S**_*i*_ = **S**_1_ for all *i*, and standard textbook methods that assume independently and identically distributed data may be used instead of meta-analytic methods. In this case, in the absence of covariates, the sample covariance matrix can be used as the estimate of the total variance (**S**_*i*_ + ***Σ***) of each study; we can also justify this estimate as the restricted maximum likelihood (REML) estimate of (**S**_*i*_ + ***Σ***). Hence, we will examine the properties of the proposed method assuming that there are no covariates, all studies have the same within-study covariance matrix (and provide all outcomes) and where the estimated total variance for each study is the sample covariance matrix, which we will denote by **V**.

It is straightforward to show that *H*^2^ = 1 in this simplified situation. Because *N* = *nd* when all studies provide all outcomes and *p* = *d* for a multivariate meta-analysis where there are no covariate effects, (8) becomes

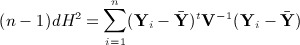


where, because all studies have the same variance structure, 
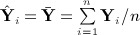
. Then, using ‘tr’ to denote the trace operator, we have

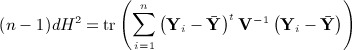


so that, interchanging the trace and the summation and using the properties of the trace operator,

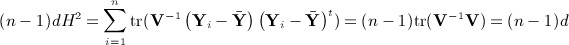


so that *H*^2^ = 1 as stated. Hence, for example, when using (14) to calculate confidence intervals, the only difference between the proposed and conventional methods is that the proposed method uses a *t*, rather than a standard normal, distribution when making inferences. We are therefore reassured that the proposed method provides appropriate inference for this simple special case. This is also the case for the other types of inferences described previously, where the only impact of the refined method is to use alternative distributions for making inferences when *H*^2^ = 1. These alternative distributions are similar to the conventional ones, but the refined method′s distributions result in larger confidence intervals and confidence regions. This is appropriate because the conventional methodology does not take into account the uncertainty in the between-study variance. However, the degrees of freedom of the *t* distribution used to make inferences are (*N* − *d*). Hence, the standard textbook result for normal identically distributed data in this simple scenario is only recovered exactly in the univariate case.

To summarise, all the usual inferences using the conventional method may be made using the proposed refined method. We found that the refined method performs better than the conventional one univariately, but like the conventional method, it takes all variance components as known and is also therefore not exact. In the next section, we perform a simulation study to investigate whether the refined method also performs better multivariately. Because the conventional method is ensured to perform well in large samples because it is justifiable asymptotically, the simulation study will focus on the common situation where there is just a handful of studies. It is therefore in this type situation where the proposed refined method has the most potential to provide a useful alternative.

## 4. Simulation study

In order to compare the proposed method with the conventional approach, the same type of procedure and 25 sets of parameter values used in the simulation study by Jackson *et al.* 17 were also used here. Hence, the simulation study only investigates bivariate meta-analyses and meta-regressions; investigating higher dimensions could form the subject of further investigation. For each simulation, two sets of *n* within-study variances were simulated from 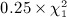 but where values outside the range [0.009, 0.6] were discarded. These two sets of within-study variances were then ranked (so that ‘larger’ studies provide more accurate estimates for both outcomes), and the first study was taken to have the largest pair of simulated variances, and so on, until the last study had the smallest pair of simulated within-study variances. New within-study variances were simulated for every meta-analysis in the simulation study. Study outcomes were simulated using means of zero, although this choice is immaterial. Between-study variances of 0, 0.024 and 0.168 were used because these values correspond to *I*^2^ statistics of 0, 0.3 and 0.75, respectively 17. Within (for all studies) and between study correlations of 0, 0.7 and 0.95 were used. The parameter values used for the variance components in the simulation study are shown in Table 1. All initial simulations involved complete data for both outcomes, but missing data for one outcome was subsequently considered.

**Table I:** Parameter values used in the simulation study.

Run	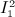	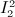	*κ*	*ρ*	Run	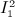	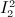	*κ*	*ρ*
1	0	0	0	0	2	0	0.3	0	0
3	0	0.75	0	0	4	0.3	0	0	0
5	0.3	0.3	0	0	6	0.3	0.75	0	0
7	0.75	0	0	0	8	0.75	0.3	0	0
9	0.75	0.75	0	0	10	0.3	0.3	0.7	0.7
11	0.3	0.75	0.7	0.7	12	0.75	0.3	0.7	0.7
13	0.75	0.75	0.7	0.7	14	0.3	0.3	0.95	0.95
15	0.3	0.75	0.95	0.95	16	0.75	0.3	0.95	0.95
17	0.75	0.75	0.95	0.95	18	0.3	0.3	0.7	0
19	0.3	0.75	0.7	0	20	0.75	0.3	0.7	0
21	0.75	0.75	0.7	0	22	0.3	0.3	0.95	0
23	0.3	0.75	0.95	0	24	0.75	0.3	0.95	0
25	0.75	0.75	0.95	0					

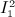
 and 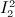 denote the *I*^2^ statistics in the first and second outcomes, respectively, and *κ* and *ρ* denote the between and within-study correlations.

### 4.1. Results using the multivariate method of moments

We initially estimated the between-study covariance matrix for each simulated dataset using the multivariate method of moments 17, but we examined alternative estimators in the succeeding text. All the accounts of the univariate refined method for meta-analysis 4–6 describe the corresponding univariate method of DerSimonian and Laird 8, so this estimator provides an obvious extension of their methodology. Having estimated the between study covariance matrices, inferences for the pooled effects can be made using the conventional 17 and the proposed refined methods so that they can be compared. We computed nominal 95% confidence intervals using both methods, and we used the proportion of intervals that contain the true values of zero to determine which method performed best.

Because both methods are justifiable asymptotically, it is in meta-analyses with small numbers of studies that the proposed method has the potential to offer an advantage. We therefore used *n* = 2,3,4,5 and then *n* = 10 to include the scenario considered previously 17. We created 100 000 simulated datasets in each run to accurately determine the coverage probabilities, and we used different random seeds to generate datasets for the two methods, in order to produce independent simulated datasets so that the Monte Carlo error of the differences in the coverage probabilities can easily be obtained. For example, because the nominal coverage probability is 0.95, differences in coverage greater than 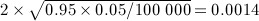 provide evidence of a genuine difference from this fixed value, and coverage probabilities between the two methods that differ by around 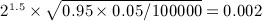 provide evidence of different probabilities; these values may be increased to account for multiple testing.

**Figure 1:**
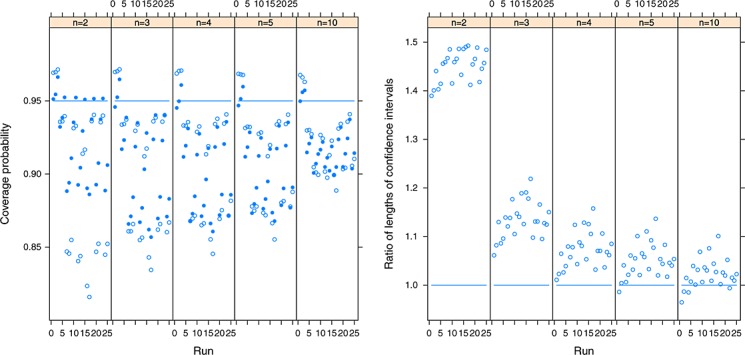
Left panel: the coverage probabilities of the proposed refined (solid points) and conventional (hollow points) methods for the first outcome, where the nominal coverage is 95% and where the between-study covariance matrix has been estimated using the method of moments. Right panel: the ratio of the average lengths of nominal 95% confidence intervals using the refined and conventional procedures, where ratios greater than one indicate that the refined method provides greater average confidence interval lengths. A total of 100 000 simulations were used for each simulation run.

For the complete data simulations, the coverage probabilities of the nominal 95% confidence intervals for the first outcome are shown in the left panel of Figure 1 (the results for the second outcome can be obtained by symmetry) where the proposed refined method is shown using solid points, and the conventional method is shown using hollow points. The proposed method appears to be an improvement over the conventional method, in that it does not result in some of the very low coverage probabilities when heterogeneity is present and it also appears to reduce the conservative nature of the conventional method when the first outcome is homogenous. In all instances, the conventional method produces the intervals with coverage probabilities that differ the most from 0.95 and in both directions. This improvement is clear for *n* = 2,3,4,5 and is still perceptible for *n* = 10, but when the sample becomes this large, the two methods perform very similarly and more satisfactorily. The approximate nature of both methods is evident from Figure 1, but of the two possibilities, the proposed method appears to be preferable.

In the right panel of Figure 1, we show the ratio of the average lengths of the 100 000 nominal 95% confidence intervals resulting from the refined and conventional procedures, where ratios greater than one indicate that the refined method provides greater average confidence interval lengths than the conventional procedure. From this figure, we see that the better coverage probabilities provided by the refined method for small *n* can be explained by the longer confidence intervals it also produces. As *n* increases, the ratios of the average lengths of confidence intervals become closer to one, and interestingly, for *n* = 10, we see that the refined method results in shorter average confidence interval lengths when the data are homogenous. This is possible because the scaling factor may be less than one, and so the refined method seems to be able to counteract, to some extent, the conservative nature of fitting the random effects model when the data are homogenous. More generally however, the refined method results in longer confidence intervals than the conventional method, which is appropriate because the conventional method does not take into account the very considerable uncertainty in the between-study variance structure. However, both methods take the between-study covariance matrix as known, which explains the under-coverage of the confidence intervals when between-study heterogeneity is present.

Interestingly, the proposed method appears to perform better for *n* = 2 than *n* = 3. This can be explained by the much longer confidence intervals that the refined method produces, compared with the conventional procedure, when *n* = 2 (right hand panel of Figure 1). The ratios of the lengths of the confidence intervals in Figure 1 depend on both the distribution of *H* and the degrees of freedom of the *t* distribution. When *n* = 2, we use a *t* distribution with just two degrees of freedom. This helps to provide much longer confidence intervals than the conventional approach and hence relatively good coverage, for this case. However, for larger *n*, the refined method uses a *t* distribution with greater degrees of freedom, and so the impact of using quantiles from the *t* distribution is diminished. This explains why better coverage for *n* = 2, than *n* = 3, is obtained when using the proposed method.

### 4.2. Missing outcome data

**Figure 2:**
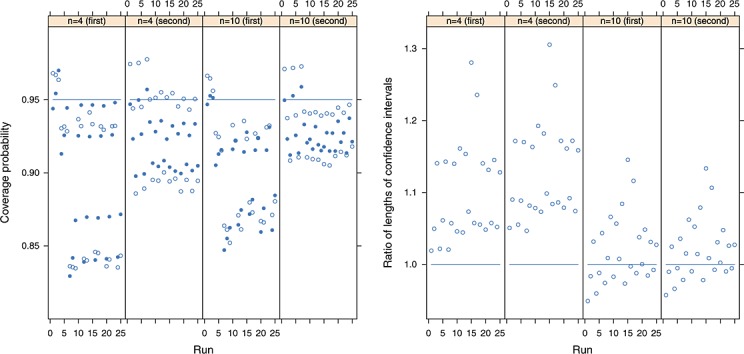
Left panel: the coverage probabilities of the proposed refined (solid points) and conventional (hollow points) methods for the first and second outcomes where half the first outcome is missing completely at random, where the nominal coverage is 95% and where the between-study covariance matrix has been estimated using the method of moments. Right panel: the ratio of the average lengths of nominal 95% confidence intervals using the refined and conventional procedures, where ratios greater than one indicate that the refined method provides greater average confidence interval lengths. A total of 100 000 simulations were used to estimate all coverage probabilities.

Multivariate meta-analysis is potentially especially useful in situations where some outcomes are missing so that all studies contribute to inferences for all outcomes because of the borrowing of strength 3 that the multivariate approach can provide. In order to investigate how the proposed refined method performs in such situations, the simulation study was repeated with *n* = 4 and *n* = 10 with half of the studies’ first outcomes missing completely at random. The results are shown in Figure 2. The proposed approach seems preferable to the conventional approach because it avoids the lowest coverage probabilities for the second (complete) outcome and because it performs similarly for the first outcome. The scaling factor is a random variable, and the ratios of the average lengths of the nominal 95% confidence intervals are not identical (but are similar) for both outcomes.

### 4.3. Alternative estimates of the between-study covariance matrix

The aforementioned simulation study provides evidence that the proposed method can provide more accurate inferences than the conventional alternative when the between-study covariance matrix is estimated using the multivariate method of moments 17. However, alternative estimators are available 3,18–20. An alterative matrix-based multivariate generalisation of DerSimonian and Laird′s univariate estimate 18,19 produced very similar results to those shown in Figures 1 and 2, and the results using this method are available in the supplementary materials that accompany the paper ‡.

**Figure 3:**
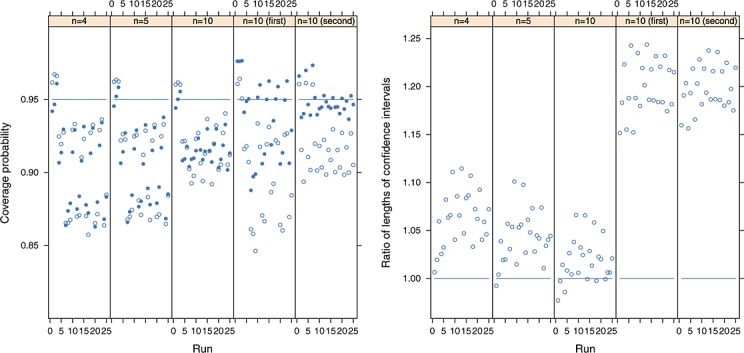
Left panel: the coverage probabilities of the proposed refined (solid points) and conventional (hollow points), where the nominal coverage is 95% *and where the between-study covariance matrix has been estimated using restricted maximum likelihood*. Right panel: the ratio of the average lengths of nominal 95% confidence intervals using the refined and conventional procedures, where ratios greater than one indicate that the refined method provides greater average confidence interval lengths. A total of 10 000 simulations were used to estimate all coverage probabilities. In each panel, the first three plots show the results for complete data and *n* = 4,5,10, and the last two plots show the results for both outcomes when *n* = 10 and half the first outcomes are missing completely at random.

Restricted maximum likelihood is the default option of the Stata and R *mvmeta* programmes 16,26, so there is particular interest in how the proposed refined method performs when used in conjunction with the REML estimator. Performing simulation studies with very small samples is problematic when using REML however, because the resulting likelihood function is often very flat, so numerical problems when attempting the numerical maximisation of this function are almost inevitable. Hence, it was decided to avoid investigating the smallest sample sizes, but the simulation study was repeated using *n* = 4,5,10 with complete data and *n* = 10 with missing data; the number of simulations was reduced to 10 000 because REML estimation is much slower, and the results are shown in Figure 3, where the first three plots in each panel show the results for complete data and the last two plots show the results where *n* = 10 and half the first outcomes are missing completely at random. The refined method appears to generally help to avoid the lowest coverage probabilities when the data are complete (most of the lowest coverage probabilities are from the conventional method), and the refined method has clearly helped to retain the nominal coverage probability when *n* = 10 and data are missing.

The considerably greater coverage probabilities of the refined method in the left panel of Figure 3 where data are incomplete can be explained by the corresponding longer average lengths of confidence intervals shown in the right panel. Comparing the REML results with incomplete data to those produced using the method of moments more closely, REML was effective in reducing (but not removing) the upward bias of the estimated between-study variance of the second (complete) outcome. This results in shorter confidence intervals, but it can be seen from Figure 2 that the method of moments’ confidence intervals using the conventional method are too short to provide 95% coverage; the conventional implementation of REML therefore appears to have suffered as a result of its better estimation of this variance component. However, the scaling provided by the refined method counteracts this, and much better inference for the average effect is obtained, especially for the second outcome.

### 4.4. Multivariate meta-regression

In order to investigate how the proposed method performs for multivariate meta-regression, we performed a further simulation study. Meta-regressions require a reasonably large number of studies to estimate covariates effects, so we used *n* = 10, with complete data, where we continued to simulate bivariate data and use the 25 sets of parameter values in Table 1 for the between-study variation. We introduced a covariate where the first, third, fifth, seventh and ninth studies provide a covariate of zero and the other studies provide a covariate of one. This covariate structure was chosen in order to mimic a covariate describing the type of study, where there are two equally likely study types. The true values of the regression parameters were taken to be one and two for the first and second outcomes, respectively. We have provided the results from this simulation study in the supplementary materials that accompany the paper, which show that the proposed method helps to avoid the largest deviations (in both directions) from the nominal 95% coverage probability and so appears preferable to the conventional approach.

To summarise, the simulation study suggests that the multivariate refined method can result in more accurate inference than the conventional method. However, the situations in which it offers such an improvement appears to depend on which estimate of the between-study covariance matrix is used. We leave the possibility of using the refined method with further estimators of the between-study covariance matrix, such as the method based on U statistics 20, as further work.

## 5. Application to examples

In this section, we apply our proposed refined method, and the conventional method, to two real examples. Both examples involve a small number of studies and so present situations where the refined method may offer a worthwhile improvement on the conventional approach. Before applying our methods to the example datasets however, we explain how to use the output from statistical software, which uses the conventional method 16,26, to produce results using the proposed method. The fact that very little extra computation is needed to apply our method increases its appeal. We used the Stata module *mvmeta* 16 to produce the all the results that follow.

### 5.1. Using standard output from statistical software to refine the conventional method

Multivariate meta-analysis packages 16,26 provide estimates, standard errors, confidence intervals and results from hypothesis tests for each parameter separately. The first step when using both the conventional and refined methods is to estimate the between-study covariance matrix. This estimation can be performed using the standard software in the usual way. The pooled estimates from the proposed and conventional methods are the same, so all that is left to do is to multiply the variance of the pooled estimates by *H*^2^ (equation (8); alternatively and equivalently, multiply the standard errors by *H*) and use a *t* distribution instead of a standard normal when making inference.

*H*^2^ is simply the ratio of the multivariate *Q*_*s*_ statistic of Jackson *et al.* 27 and (*N* − *p*), where the estimated between-study covariance matrix has been added to the within-study covariance matrices before computing *H*^2^. Standard software packages (as ‘e(Qscalar)’ and simply ‘*Q*’ in the Stata 16 and R 26 *mvmeta* packages, respectively) routinely provide the *Q*_*s*_ statistic. Having used the conventional software packages to estimate the between-study covariance matrix, the user can create total covariance matrices (as the sum of the within and the estimated between-study covariance matrices) and refit the model where the within-study covariance matrices are given as the total covariance matrices. The reported *Q*_*s*_ can then be divided by (*N* − *p*) to give *H*^2^. It is then a very simple task to multiply the variance of the pooled estimates in the conventional analysis by *H*^2^ (or their standard errors by *H*) and use a *t* distribution for making inferences, as explained in Section 3.1, and so apply the proposed method.

### 5.2. Example one

The periodontal data from Berkey *et al*. 1 have been used as an example of a bivariate meta-analysis several times 27. This example involves five studies providing the mean difference between a surgical and non-surgical procedure for treating periodontal disease. Improvements in probing depth and attachment level are the two outcomes of interest (measured in *mm* 1 year after treatment). The data are shown in Table 2, where the outcomes are the mean differences. The within-study correlations are positive as one might expect because patients who respond better to treatment in terms of probing depth also respond better in terms of attachment level.

**Table II:** Periodontal data, providing the mean difference between a surgical and non-surgical procedure for treating periodontal disease, with improvement in probing depth and improvement in attachment level as the two end outcomes of interest (measured in *mm*, 1 year after treatment).

Study	Outcome 1	Within-study variance	Outcome 2	Within-study variance	Within-study covariance
1	0.47	0.0075	− 0.32	0.0077	0.0030
2	0.20	0.0057	− 0.60	0.0008	0.0009
3	0.40	0.0021	− 0.12	0.0014	0.0007
4	0.26	0.0029	− 0.31	0.0015	0.0009
5	0.56	0.0148	− 0.39	0.0304	0.0072

The within-study variances and covariances are known so all entries of the within-study covariance matrices **S**_*i*_ are known.

#### 5.2.1. Results using the multivariate method of moments

The *H*^2^ statistic is 0.998 when using the multivariate method of moments 17 to estimate the between-study covariance matrix. Multiplying the variance of the pooled estimates by this scaling factor makes little difference, but the proposed refined method uses a *t* distribution with *N* − *p* = 10 − 2 = 8 degrees of freedom, rather than a standard normal, when computing confidence intervals and performing hypothesis tests. The pooled estimates are 0.35 and -0.34, and the conventional method provides 95% confidence intervals of (0.24, 0.46) and (-0.56, -0.12); the refined method provides 95% confidence intervals of (0.22, 0.48) and (-0.60, -0.08). A positive average treatment effect for the first outcome, and a negative average treatment effect for the second, are inferred when using both methods. However, the proposed method results in wider confidence intervals; this seems to be appropriate because the conventional method is known to provide confidence intervals that are too short to achieve the nominal coverage when heterogeneity is present 17. The 95% percentile of a *t* distribution with 8 degrees of freedom is 2.31, so because *H*^2^ is effectively 1, the effect of the refined method is to widen the 95% confidence intervals by a factor of 2.31 / 1.96 (around 18%).

#### 5.2.2. Results using the matrix-based method of moments

The *H*^2^ statistic is 0.877 when using the matrix-based method of moments 18,19. The pooled estimates are again 0.35 and -0.34, but the conventional method provides 95% confidence intervals of (0.23, 0.48) and (-0.56, -0.12); the refined method provides 95% confidence intervals of (0.21, 0.49) and (-0.58, -0.09). These results are similar to those in the previous section. The refined method widens the confidence intervals by around 10%.

#### 5.2.3. Results using restricted maximum likelihood

Here, REML was applied using the defaults of Stata′s *mvmeta* 16 so that the inverse of the entire Fisher information matrix (including the variance components) was used to compute the covariance matrix **C**. The pooled estimates are again 0.35 and -0.34, but the conventional method provides 95% confidence intervals of (0.23, 0.47) and (-0.51, -0.16); here *H*^2^ = 1.030 and the refined method provides 95% confidence intervals of (0.21, 0.50) and (-0.55, -0.13). The refined method widens the confidence intervals by around 19%.

### 5.3. Example two

Our second example considers the prognostic effect of excision repair cross-complementation group 1 protein in patients with non-small cell lung cancer who did not receive chemotherapy. High-versus-low expression of excision repair cross-complementation group 1 protein was examined in relation to event-free survival and overall survival from six studies identified by a systematic review 28. The data are shown in Table 3, with log hazard ratios and their variances reported for event-free and overall survivals as outcomes 1 and 2, respectively. We recognise that if individual patient data were available, then we could investigate more advanced statistical issues (such as competing risks and non-proportional hazards). All six studies provide an estimate for overall survival, but only three provide estimates for event-free survival. This is a situation where multivariate meta-analysis is potentially useful, for event-free survival to borrow strength from overall survival. However, the within-study correlations are unknown. A thorough sensitivity analysis, examining a wide-range of possible within-study correlations, or other strategies for handling unknown within-study correlations 3,29 should be used in such situations in practice, but here we just consider within-study correlations of 0.7 for illustrative purposes. A high within-study correlation is expected, however, because of the strong association between time to death and time to recurrence of disease.

**Table III:** Prognostic effect of excision repair cross-complementation group 1 protein data.

Study	Outcome 1	Within-study variance	Outcome 2	Within-study variance
1	0.54	0.16	0.73	0.16
2	−	−	− 0.49	0.06
3	−	−	0.52	0.20
4	− 0.04	0.10	− 0.04	0.10
5	−	−	− 1.08	0.20
6	− 0.44	0.08	− 0.62	0.06

The within-study covariances are unknown.

#### 5.3.1. Results using the multivariate method of moments

The pooled estimates are -0.14 (for event-free survival) and -0.20 (for overall survival), and the conventional method provides 95% confidence intervals of (-0.59, 0.32) and (-0.68, 0.29). No statistically significant effect is inferred. *H*^2^ = 0.836 so that the scaling factor is less than one, but the refined method uses a *t* distribution with *N* − *p* = 9 − 2 = 7 degrees of freedom when making inferences, and wider 95% confidence intervals of (-0.64, 0.37) and (-0.73, 0.34) are obtained using this method. These wider confidence intervals seem to be more appropriate than the conventional ones, which do not reflect the very considerable uncertainty in the between-study covariance matrix. The refined method widens the confidence intervals by around 10%.

#### 5.3.2. Results using the matrix-based method of moments

The *H*^2^ statistic is 0.869 when using the matrix-based method of moments 18,19. The pooled estimates are again -0.14 and -0.20, but the conventional method provides 95% confidence intervals of (-0.55, 0.28) and (-0.67, 0.28); the refined method provides 95% confidence intervals of (-0.60, 0.33) and (-0.73, 0.34). These results are similar to those in the previous section, and the refined method widens the confidence intervals by around 12%.

#### 5.3.3. Results using restricted maximum likelihood

We have also obtained very similar results using REML for this example. Here the pooled estimates are -0.13 and -0.19, and the conventional method provides 95% confidence intervals of (-0.59, 0.32) and (-0.70, 0.31). *H*^2^ = 0.783, but because a *t* distribution with 7 degrees of freedom is used for making inferences, the refined method again gives wider 95% confidence intervals of (-0.62, 0.35) and (-0.73, 0.35). The refined method widens the confidence intervals by around 7%.

## 6. Discussion

We have proposed a multivariate generalisation of the refined method for univariate meta-analysis. Just as in the univariate case, the refined method can provide more accurate inferences than the conventional alternative in situations where there is not a very large number of studies to justify the conventional asymptotic approximations. However, the refined method also takes the variance components as known and so is also an approximation. Methods that take into account the uncertainty in the between-study covariance matrix are available 3 but are much more computationally intensive than the proposed approach, especially in high dimensions. When using methods based on maximum likelihood, another possible improvement on the conventional approach is to invert the entire Fisher information matrix (including variance components) to obtain the covariance matrix of the pooled effects. We can also apply the refined method when inverting the entire Fisher information matrix in this way, by scaling the resulting standard errors by *H* as we propose, and as we show for our two examples in Section 5 where REML is used. A Bayesian approach would fully take into account the uncertainty in the between-study variance components but would come at the price of issues concerning prior sensitivity.

Because the conventional approach is justified asymptotically, the proposed refined method cannot offer much advantage in situations where there are very many studies. Hence, it is in the common situation where there are relatively few studies, such as in our example datasets and simulation studies, that we suggest the proposed approach should be considered. However with few studies, the point estimation (which the proposed method does not affect) is especially fragile as shown in an example in Jackson *et al.* 3 where the between-study correlation is poorly estimated as -1 using the method of moments and +1 using REML, which leads to considerably different pooled estimates. Hence all results from multivariate meta-analyses should be interpreted cautiously when few studies contribute to the analysis and in application the data should be carefully inspected for any apparently anomalous, outlying or influential values. A comparison of the results from multivariate meta-analyses with their univariate counterparts is perhaps especially important when there are few studies. The statistician should give a great deal of consideration to whether or not the more sophisticated methods for multivariate meta-analysis should be applied to their examples, but in situations where this is to be attempted, and the number of studies is small, we suggest that our refined method should, at the very least, be considered as an alternative to the conventional one. The very cautiously minded statistician could also constrain the scaling factor *H*^2^ to be more than or equal to one so that the proposed method cannot result in shorter confidence intervals and more significant hypothesis tests, in situations where such conservatism is warranted.

One can easily compute our proposal using standard output from multivariate meta-analysis software. This is an advantage of our proposed method. Hence, statisticians may use whichever multivariate meta-analysis programme they are familiar with to implement our method. Statisticians are also free to use their preferred estimator of the between-study covariance matrix in conjunction with our proposal. One can also use our method in situations where structured between-study covariance matrices, or even a fixed effect model, are assumed; once the structured between-study covariance matrix has been estimated (or ignored in a fixed effect model), one can apply our method by scaling variances by *H*^2^ and using a *t* distribution. Hence our proposed method is widely applicable and accessible to those using multivariate meta-analysis.

## Supporting Information

Supporting info item
