# Supplementary material for: A refined method for multivariate meta-analysis and meta-regression
Source: Stat Med. 2013 Aug 29;33(4):541–54. doi: 10.1002/sim.5957 (PMC4285306; doi:10.1002/sim.5957)
Supplement: Supplementary file 1 — Supporting info item [file sim0033-0541-sd1.pdf]

Research Article

Statistics in Medicine

Received XXXX

(www.interscience.wiley.com) DOI: 10.1002/sim.0000

Supplementary materials for “A refined method for multivariate meta-analysis and meta-regression”

Dan Jackson* and Richard D Riley†

1. Simulation Study results for multivariate meta-analysis using the matrix based method of moments

A matrix based multivariate method of moments has recently been proposed [1, 2]. Figures 1 and 2 below show the results, as in figures 1 and 2 of the main paper, but using this alternative method. Comparing these two figures to figures 1 and 2 of the main paper shows that very similar results are obtained.

*MRC Biostatistics Unit, Cambridge, UK.
†University of Birmingham
*Correspondence to: daniel.jackson@mrc-bsu.cam.ac.uk

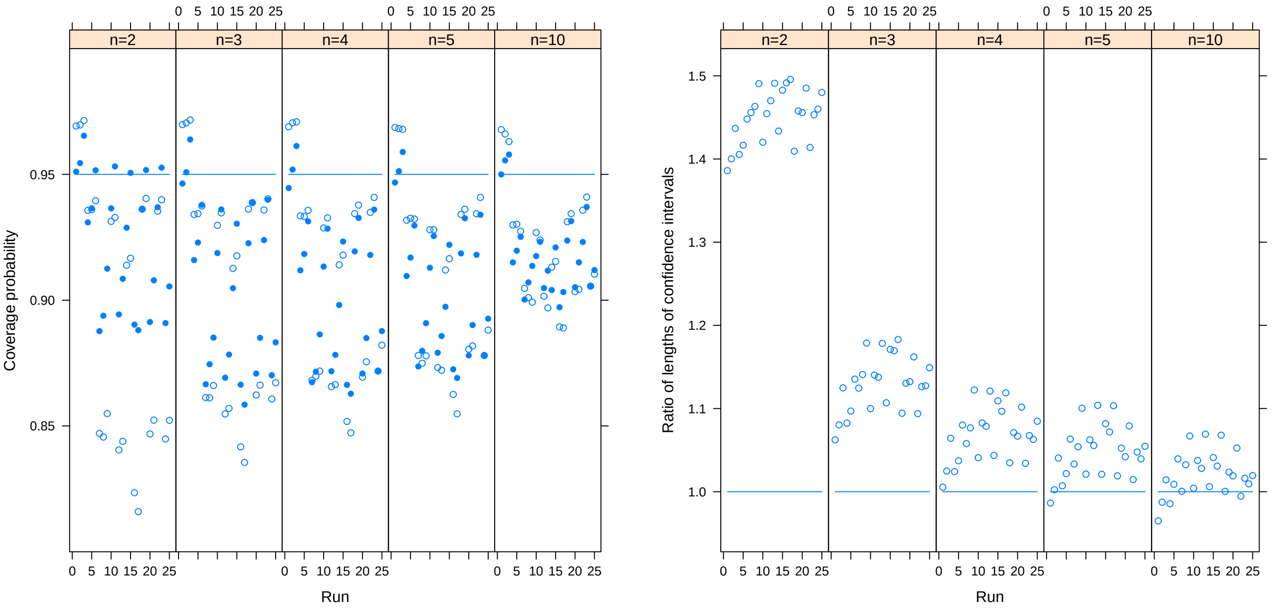

Figure 1. Left panel: The coverage probabilities of the proposed refined (solid points) and conventional (hollow points) methods for the first outcome, where the nominal coverage is 95% and where the between-study covariance matrix has been estimated using the *matrix based* method of moments. Right panel: The ratio of the average lengths of nominal 95% confidence intervals using the refined and conventional procedures, where ratios greater than one indicate that the refined method provides greater average confidence interval lengths. 100,000 simulations were used for each simulation run.

Copyright © 2009 John Wiley & Sons, Ltd.

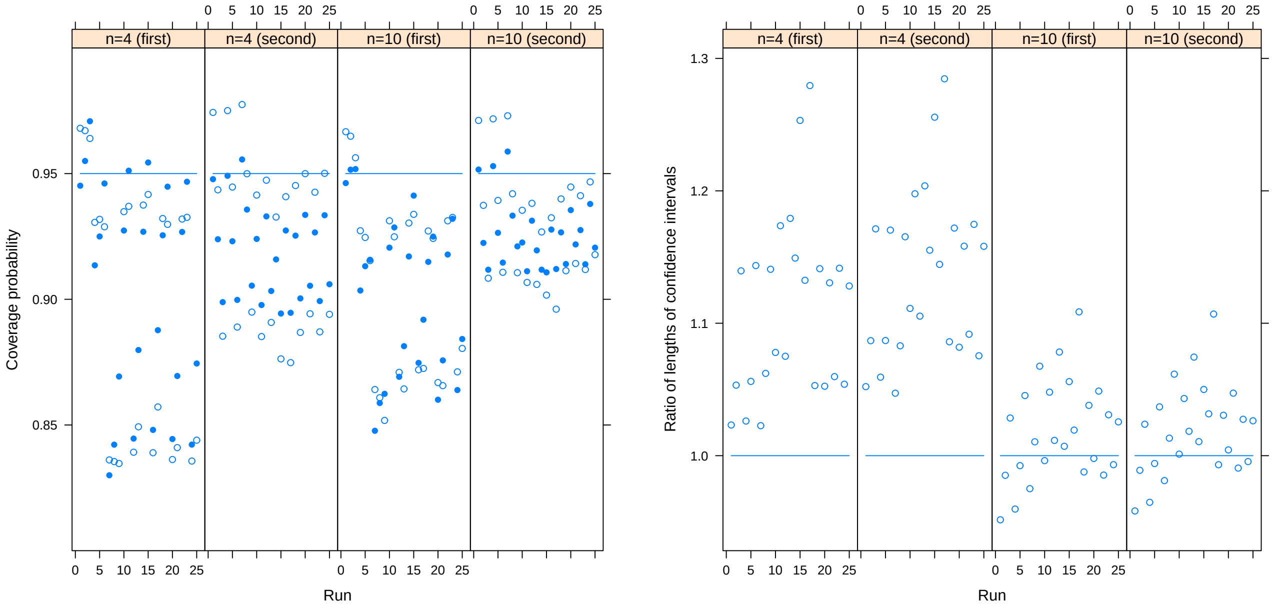

Figure 2. Left panel: The coverage probabilities of the proposed refined (solid points) and conventional (hollow points) methods for the first and second outcomes where half the first outcome is missing completely at random, where the nominal coverage is 95% and where the between-study covariance matrix has been estimated using the *matrix based* method of moments. Right panel: The ratio of the average lengths of nominal 95% confidence intervals using the refined and conventional procedures, where ratios greater than one indicate that the refined method provides greater average confidence interval lengths. 100,000 simulations were used to estimate all coverage probabilities.

2. Simulation Study results for multivariate meta-regression

In order to investigate how the proposed method performs for multivariate meta-regression, a further simulation study was performed. Meta-regressions require a reasonably large number of studies to estimate covariates effects, so we used $n = 10$, with complete data, where we continued to simulate bivariate data and use the 25 sets of parameter values in table 1 for the between-study variation. We introduced a covariate where the 1st, 3rd, 5th, 7th and 9th studies provide a covariate of 0 and the other studies provide a covariate of 1. This covariate structure was chosen in order to mimic a covariate describing the type of study, where there are two equally likely study types. The true values of the regression parameters were taken to be one and two for the first and second outcomes respectively.

The results are shown for all three methods used in the main paper (the method of moments [3], the matrix based method of moments [2], and REML) in figure 3, where we show the results for the coverage probabilities, and lengths, of the confidence intervals for the two covariate effect regression parameters. In figure 3, '1st' and '2nd' indicate that the results correspond to the covariate effect for the first and second outcomes respectively. Figure 3 shows that the proposed method helps to avoid the largest deviations (in both directions) from the nominal 95% coverage probability and so appears preferable. The proposed method generally provides longer confidence intervals, which explains the better coverage probabilities and is appropriate because the conventional approach does not take the uncertainty in the between-study variance structure into account.

References

1. Chen H, Manning AK, Dupuis, J. A method of moments estimator for random effect multivariate meta-analysis. *Biometrics* 2012; 64: 1278–1284
2. Jackson D, White IR, Riley RD. A matrix based method of moments for fitting the multivariate random effects model for meta-analysis and meta-regression. *The Biometrical Journal* 2013; 55: 231-245
3. Jackson D, White IR, Thompson, SG. Extending DerSimonian and Laird's methodology to perform multivariate random effects meta-analyses. *Statistics in Medicine* 2010; 29: 1282–1297.

Copyright © 2009 John Wiley & Sons, Ltd.

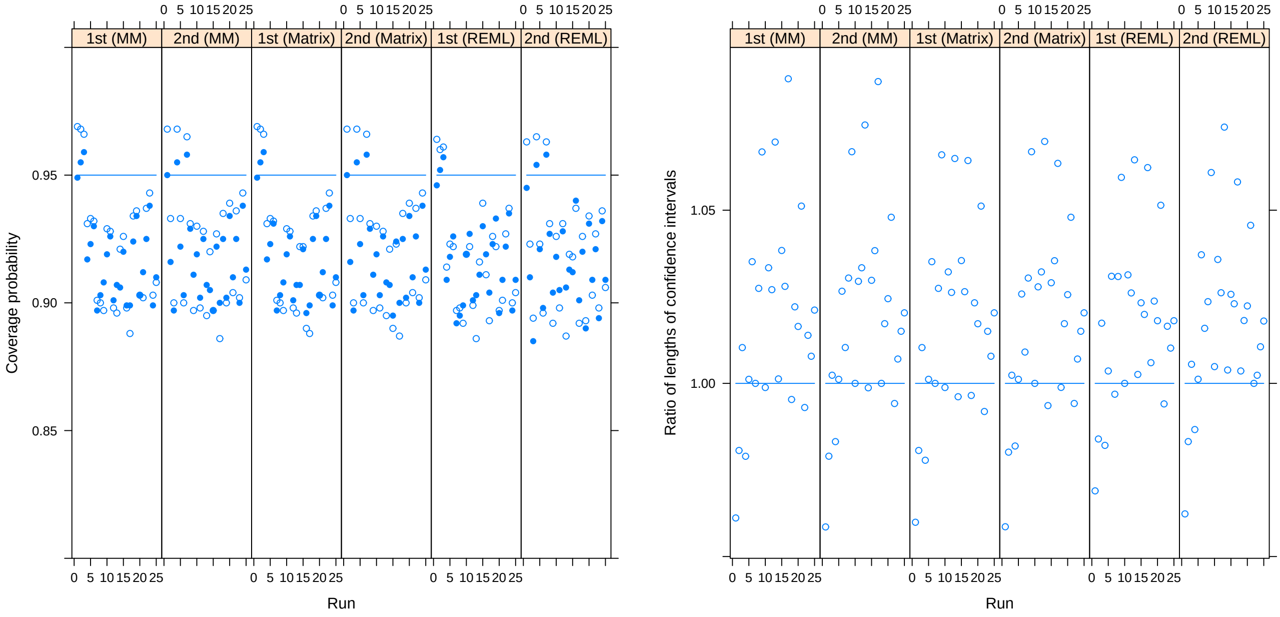

Figure 3. Left panel: The coverage probabilities of the proposed refined (solid points) and conventional (hollow points) methods, where the nominal coverage is 95% and meta-regression models are used to estimate the covariate effect for both outcomes. Right panel: The ratio of the average lengths of nominal 95% confidence intervals using the refined and conventional procedures, where ratios greater than one indicate that the refined method provides greater average confidence interval lengths. 100,000 simulations were used for each simulation run when using the two method of moments and 10,000 simulations were used for REML.

Copyright © 2009 John Wiley & Sons, Ltd.
